# Importance of multimodal resident education curriculum for general surgeons: perspectives of trainers and trainees

**DOI:** 10.1186/s12909-024-05515-x

**Published:** 2024-05-10

**Authors:** Jeeyeon Lee, Hyung Jun Kwon, Soo Yeon Park, Jin Hyang Jung

**Affiliations:** Department of Surgery, Kyungpook National University Chilgok Hospital, Hoguk-Ro 807, Buk-Gu, 41404 Daegu, Republic of Korea

**Keywords:** Surgeon, Residency, Education, Multimodal, Curriculum

## Abstract

**Purpose:**

Satisfaction should be prioritized to maximize the value of education for trainees. This study was conducted with professors, fellows, and surgical residents in the Department of general surgery (GS) to evaluate the importance of various educational modules to surgical residents.

**Methods:**

A questionnaire was administered to professors (*n *= 28), fellows (*n* = 8), and surgical residents (*n* = 14), and the responses of the three groups were compared. Four different categories of educational curricula were considered: instructor-led training, clinical education, self-paced learning, and hands-on training.

**Results:**

The majority of surgeons regarded attending scrubs as the most important educational module in the training of surgical residents. However, while professors identified assisting operators by participating in surgery as the most important, residents assessed the laparoscopic training module with animal models as the most beneficial.

**Conclusions:**

The best educational training course for surgical residents was hands-on training, which would provide them with several opportunities to operate and perform surgical procedures themselves.

**Supplementary Information:**

The online version contains supplementary material available at 10.1186/s12909-024-05515-x.

## Introduction

Continuous, lifelong medical education should be provided because it is essential to training qualified specialists in every medical field [1]. Educational periods may be categorized as medical college, postgraduate, and specialist education. The residency training program is the most important part of postgraduate education. However, departments of general surgery (GS) in the United States and South Korea are seeing decreased demand for surgical residencies [2–4].

The decreasing demand for residencies in GS is critical because it results in a lack of specialists. The reasons for this demand reduction vary and include the fatigue accumulated from emergency surgeries, the deterioration of their quality of life, and the frequent stressful situations caused by handling vital organs [5–7]. Nevertheless, many physicians continue to choose surgical residency and might feel enthusiastic when they succeed in performing surgery, thus saving patients’ lives and improving patients’ odds of survival. Although a majority of surgical applicants apply with this intent, they experience frustration and often regret their decision when faced with the harsh reality of the field. In light of these problems, surgical residency training should pay attention to the mental and physical well-being of residents. In particular, unlike the trainers’ generation, residents of the twenty-first century prioritize work-life balance, technological proficiency, adaptability to pandemic situations, diversity, and inclusion [8–11]. These factors have gained even greater emphasis following the coronavirus pandemic. Given that residents in Korea tend to avoid challenging but essential medical care, motivation becomes even more critical for those applying to GS. Educational programs that fail to consider these specific characteristics may lead to decreased achievement and efficiency.

Experts agree that instructors must consider trainees’ satisfaction to maximize educational impact [12, 13]. Each qualified specialist’s training involves numerous educational training modules, which should be accessible and tailored to the demands of surgery. Instructors should determine how to increase educational impact by verifying the degree of satisfaction of surgical residents and evaluating the efficacy of the educational curriculum. Educational programs are generally divided into instructor-led training (ILT) and self-paced learning (SPL), and the program should be structured to balance these two areas equally [14–18]. Moreover, in the education of doctors, each resident must receive not only theoretical education but also more detailed programs related to clinical and technical skills.

In this study, by evaluating the responses of professors, fellows, and surgical residents in a department of GS regarding trainee satisfaction and the importance of educational training modules, we identified the most appropriate educational curriculum for residents to establish better training courses toward cultivating specialists with higher qualifications.

## Methods

The educational curricula for surgical residents were organized by the Education Committee of the department of surgery at Kyungpook National University Hospital, Daegu, Republic of Korea. The categories of educational curricula were classified into ILT, clinical education, SPL, hands-on training, and detailed training courses (Table 1, Supplementary Fig. 1). ILT is defined as a training and learning program provided by instructors or teachers, whereas SPL is a student-driven learning design [18–20].

**Table 1:** Education curriculum for surgical residents

Category	No	Curriculum	Programs
Instructor-led training (ILT)	1	General lectures	General lectures about each organ
	2	External lectures	Lecture by professors of other departments such as respiratory medicine, emergency medicine, endocrinology, anesthesiology, and infectious medicine
	3	Special lectures	Specific lectures about each surgical part
	4	Surgical grand round	Educational conference for medical students, interns, surgical residents, and all faculties
	5	Conference	Participating in regular conferences in each division
Clinical education	6	Attending scrubs	Assisting or performing the surgery
	7	Outpatient clinic	Observation at outpatient clinic
	8	Inpatient clinic	Care for patients admitted and rounds of wards
Self-paced learning (SPL)	9	Resident journal club	Residents who meet regularly to critically evaluate recent articles with literature review
	10	Oral presentation	Oral presentation at annual meeting of Korean Surgical Society
	11	Collecting data and writing article	Data collection, analysis, and selection, and writing articles
	12	Manual for surgical residents	Practical guidelines on the basics of operative and perioperative management
Hands-on training	13	Ultrasound training module	Residency training for the basic concepts in physics, instrumentation and scanning techniques, and clinical application
	14	Animal laparoscopy training module	Animal laparoscopy training module in training center outside
	15	Dry-lab laparoscopy training module	In-hospital laparoscopy training module

The importance of different categories of resident education was assessed on the basis of a cross-sectional questionnaire proffered via e-mails or text messages. Professors, fellows, and surgical residents responded to the survey. Academic faculty and clinical specialists comprised the group of professors. Fellows were defined as specialists who had completed their residency within the immediately preceding two years. The training system for medical residents in Korea consists of a three-year format, and accordingly, surgical residents consisted of first-year, second-year, and third-year residents following the curriculum of the Korean Surgical Society. All the professors and fellows, who have successfully completed their residency and obtained specialist qualifications, were regarded as educators. And only residents in their first to third years of training were considered as trainees.

The questionnaire comprised nine items, including the level of the surgeons; their self-estimated daily working hours; and the time they estimated to have devoted to the education of residents in a week, excluding routine jobs (Supplementary Table 1). The top three important education curricula among the 15 training courses constituting the educational curricula were further classified into the four categories.

Each complete response collected from professors, fellows, and residents was analyzed and presented in the form of bar graphs for convenient comparison.

## Results

### Sociodemographic characteristics

In total, 50 surgeons responded to the questionnaires. Six of the respondents repeated their submissions because of incomplete forms. The mean ages of the groups of professors (*n* = 28), fellows (*n* = 8), and residents (*n* = 14) were 47.3 years (SD, ± 15.1), 33.3 years (SD, ± 2.3), and 31.0 years (SD, ± 3.3), respectively.

Professors specialized in breast/thyroid (n = 7, 25.0%), colorectal (*n* = 6, 21.4%), upper gastrointestinal (*n* = 5, 17.9%), vascular (*n* = 4, 14.3%), hepato-bilio-pancreatic (*n* = 3, 10.7%), pediatric (*n* = 1, 3.6%), trauma (n = 1, 3.6%), and critical care (*n* = 1, 3.6%) surgeries. Fellows majored in vascular (*n* = 3, 37.5%), colorectal (*n* = 2, 25.0%), upper gastrointestinal (*n* = 1, 12.5%), breast/thyroid (*n* = 1, 12.5%), and critical care (*n* = 1, 12.5%) surgeries. Fourteen residents from the first year (*n* = 2, 14.3%), second year (*n* = 4, 28.6%), and third year (*n* = 8, 57.1%) participated (Supplementary Table 3).

Self-estimated daily working time and time devoted to the education of residents in a week.

Professors responded that they worked 8 h (*n* = 8, 28.6%), 10 h (*n* = 13, 46.4%), and more than 12 h (*n* = 7, 25.0%) per day. However, a majority of fellows (*n* = 6, 75.0%) and residents (*n* = 12, 85.7%) responded that they worked for more than 12 h per day. Professors and residents spent approximately 2 h per week on the education of surgical residents, excluding routine jobs (Supplementary Table 2).

Most helpful educational category for the training of surgical residents.

**Fig. 1:**
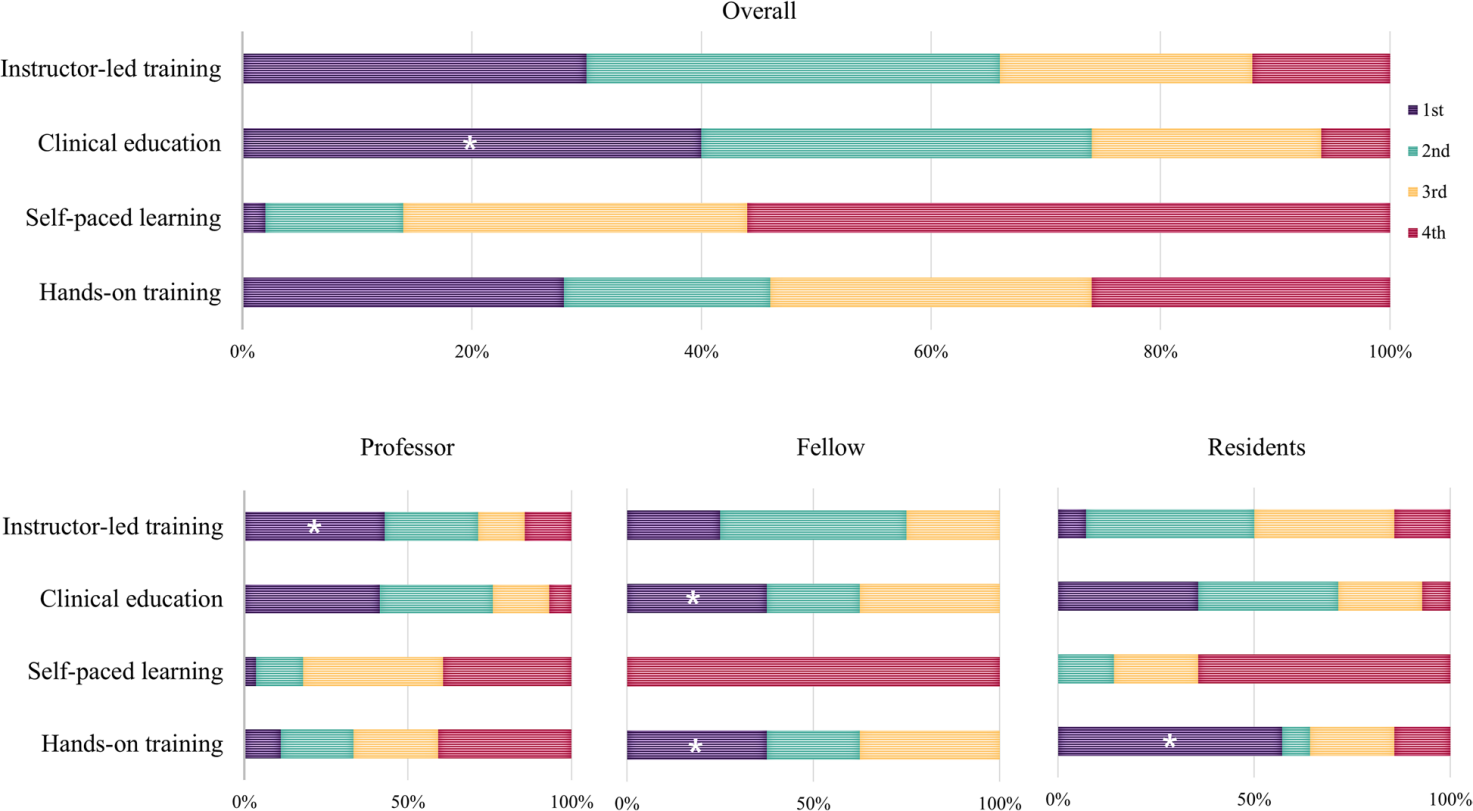
Importance ratings of four major educational categories for surgical residency training indicate that clinical education was considered the most important (*) curriculum overall. However, each group, including professors, fellows, and residents, judged different curricula to be the most important

Of the four different educational categories, clinical education was regarded as the most helpful educational course for surgical residents by all groups. A marginal majority of professors (by a slight margin) regarded the ILT course as the most helpful to education. While fellows selected clinical education and SPL as the most and least helpful courses, surgical residents identified hands-on training and SPL as the most and least helpful courses, respectively (Fig. 1).

### Top three important educational curricula for surgical residents

The identification of the three most important curricula among the 15 training courses covered in the four categories was performed by way of a featured survey question. As shown in Table 2, each group selected attending scrubs as the most important program among the three most important courses.

**Table 2:** Importance rating of educational curricula, as considered to be top threes among 15 training courses

Category	No	Curriculum	Professors (*n* = 28)	Fellows (*n* = 8)	Residents (*n* = 14)	Overall (*n* = 50)
Instructor-led training (ILT)	1	General lectures	6.0%	16.7%	14.3%	10.0%
	2	External lectures	6.0%	12.5%	4.8%	6.7%
	3	Special lectures	8.3%	0.0%	7.1%	6.7%
	4	Surgical grand round	4.8%	4.2%	2.4%	4.0%
	5	Conference	8.3%	0.0%	0.0%	4.7%
Clinical education	6	Attending scrubs	22.6%	20.8%	23.8%	22.7%
	7	Outpatient clinic	2.4%	0.0%	2.4%	2.0%
	8	Inpatient clinic	13.1%	8.3%	9.5%	10.7%
Self-paced learning (SPL)	9	Resident journal club	6.0%	4.2%	0.0%	4.0%
	10	Oral presentation	2.4%	0.0%	7.1%	4.0%
	11	Collecting data and writing article	1.2%	0.0%	0.0%	0.7%
	12	Manual for surgical residents	0.0%	4.2%	0.0%	0.7%
Hands-on training	13	Ultrasound training module	2.4%	0.0%	4.8%	2.7%
	14	Animal laparoscopy training module	7.1%	16.7%	11.9%	10.0%
	15	Dry-lab laparoscopy training module	9.5%	12.5%	11.9%	10.7%

### Importance of training courses in the ILT category for the training of surgical residents

**Fig. 2:**
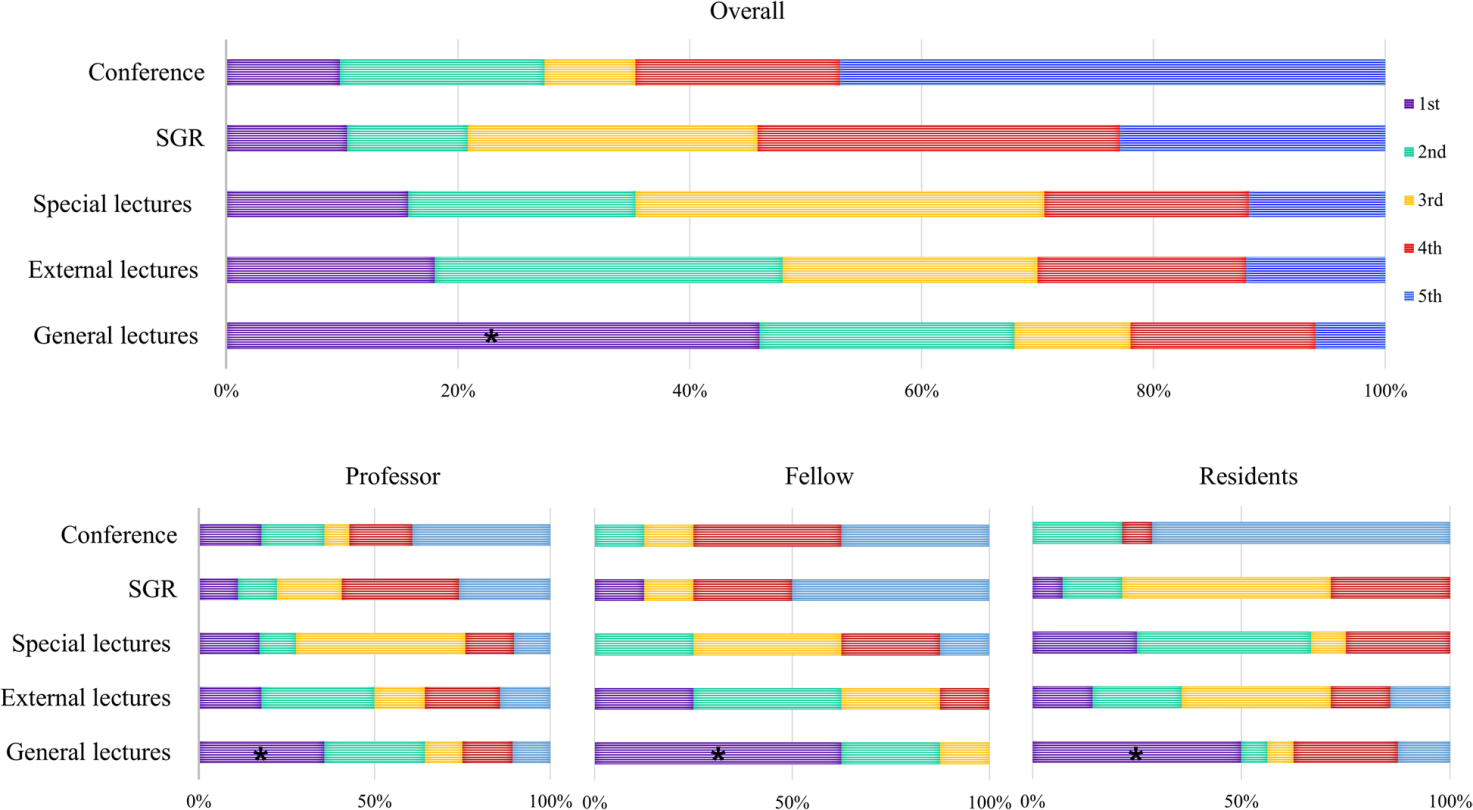
Importance ratings of instructor-led training (ILT) courses for surgical residency training indicate that general lectures were considered the most important (*) part of the curriculum overall. All groups consistently judged general lectures to be the most important educational program

All the surgeons selected general lectures by professors as the most important curriculum in the ILT category for the training of surgical residents. Notably, fellows and residents considered this curriculum the most important course to a greater extent than professors did. By contrast, professors and residents considered residents’ participation in regular in-hospital conferences the least important in resident training, whereas fellows considered the Surgical Grand Rounds as less important than other curricula (Fig. 2).

Importance of the training course in the clinical education category of surgical residents’ training.

**Fig. 3:**
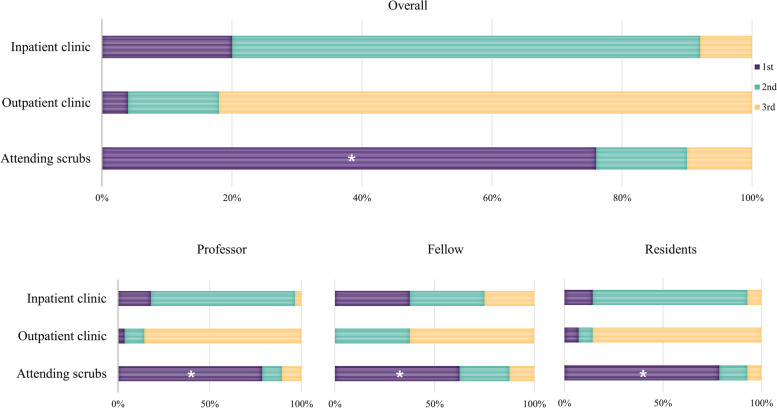
Importance ratings of courses within the clinical education category for surgical resident training indicate that attending scrubs are considered the most important, both overall and by each group

All groups responded that attending scrubs was the most important training method in clinical education. While the professor and resident groups ranked attending inpatient and outpatient clinics as second and third in importance, respectively, the fellow group rated the clinics with a similar degree of importance in clinical education (Fig. 3).

### Importance of the training course in the SPL category for the training of surgical residents

**Fig. 4:**
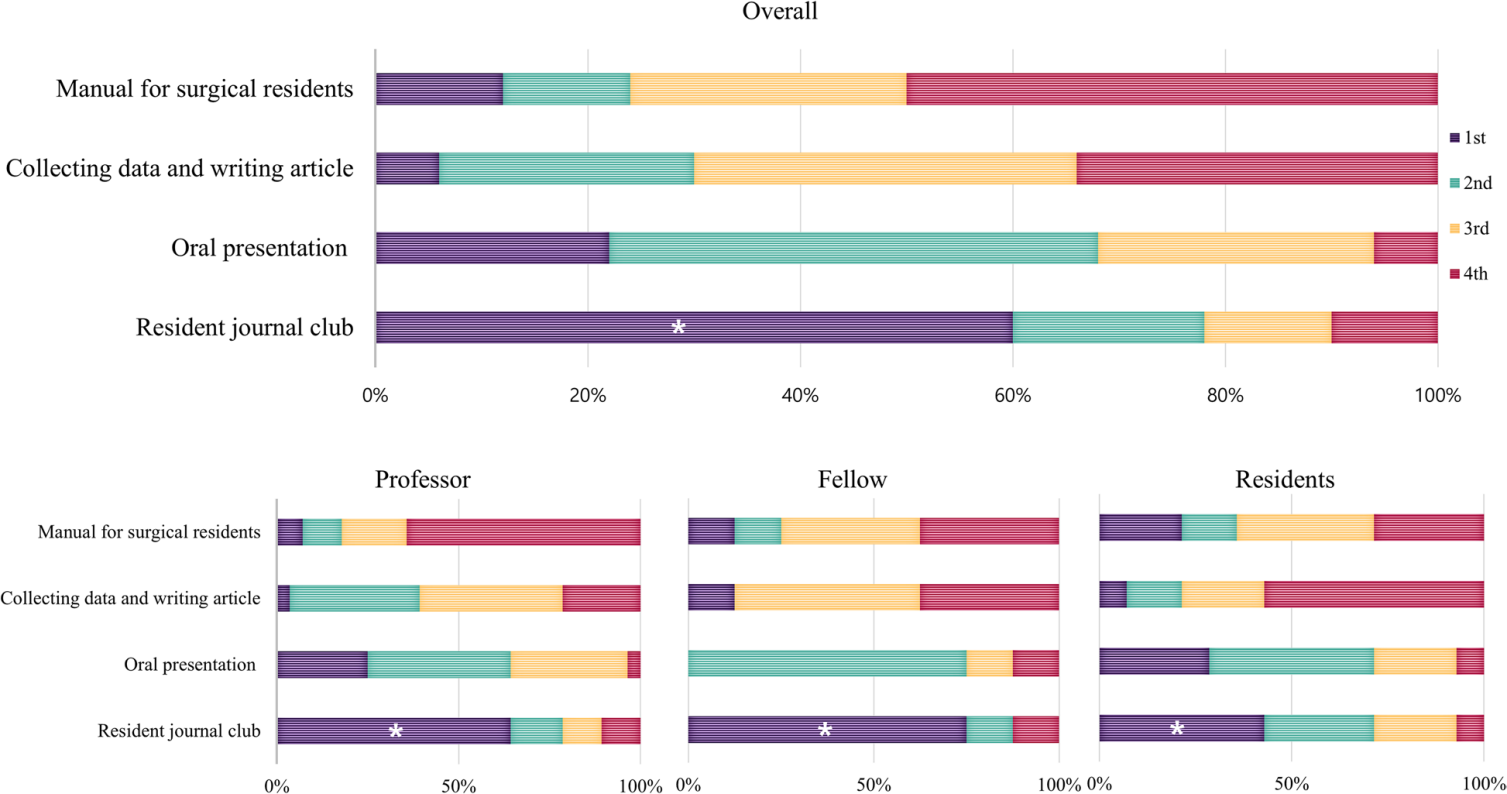
Importance ratings of self-paced learning (SPL) courses for surgical residency training indicate that resident journal club was considered the most important (*) part of the curriculum overall. And all groups consistently judged resident journal club to be the most important educational program

All groups considered the regular journal club the most important training course in the SPL category. While the professor and fellow groups considered the manual for surgical residents less important than other curricula, residents regarded it as the third-most important course. Surgical residents considered collecting data and writing articles the least important (Fig. 4).

### Importance of the training course in the hands-on training category for surgical residents’ training

**Fig. 5:**
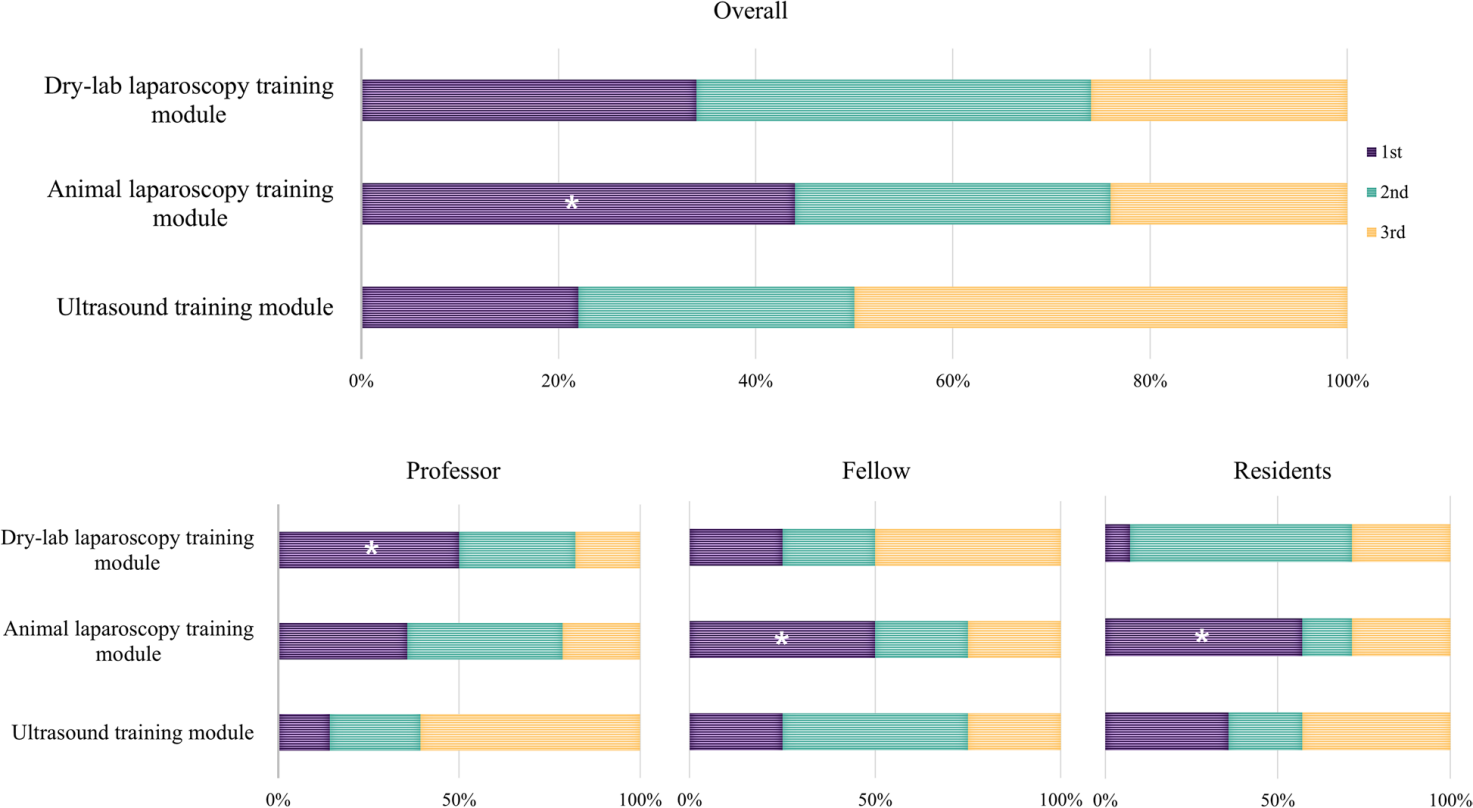
Importance rating of courses within the hands-on training category for the training of surgical residents. While the professor group judged that dry lab laparoscopy training module was the most important (*), the fellow and resident groups judged the animal laparoscopy training module to be more important

In the hands-on category, the dry-lab laparoscopy training module within the hospital was deemed by professors as the most important training course for surgical residents, whereas the laparoscopic training module in the professional education center with animal models was perceived as most important by fellows and residents (Fig. 5).

## Discussion

This study revealed that trainers and trainees differ in their criteria for evaluating the importance of educational components, with surgical residents particularly interested in hands-on training. Halsted emphasized the significance of clinical and practical training in the education of American surgeons [21]. Similarly, current GS professors and residents place a high value on practical and technical education. However, stringent ethical standards present challenges for residents practicing directly on cadavers or patients [22–26]. Consequently, professors typically offer thorough theoretical training before introducing practical skills to mitigate potential complications. The contemporary environment is causing divergent approaches between trainers and trainees. If an education system that closely simulates human conditions is developed, and a system where trainees can freely receive education within ethical boundaries is established, it could become the most efficient approach to education.

A series of educational courses must be provided to medical students, interns, residents, and fellows to ensure medical specialists’ optimal training. Although these training courses may be delivered at various levels depending on the knowledge and needs of trainees, instructors have predominantly solely developed the ILT curriculum. However, the actual effectiveness of training courses may differ from the instructors’ expectations; furthermore, considering the satisfaction of the trainees while planning the courses could improve the learning experience [12, 13, 27].

When designing curricula, it is crucial to consider that surgical residents apply for residencies because they are interested in surgery and are aspired to be surgeons. Some professors focus on teaching academic theory, while others contend that mastering the art of thesis writing is the most critical aspect of education. Nonetheless, the most basic desire of surgeons must be considered. In our current study, we found that the opinions of professors, fellows, and residents regarding the most important training courses for surgical residents were similar.

While the group of professors believed that watching many standardized surgeries is the most important training course needed by surgical residents to participate and assist in surgeries, residents considered having practical operating experience as the most important. Therefore, residents regarded the laparoscopic training module in the professional institute with animal models as the most important curriculum in the training of surgical residents. The responses from the group of fellows, who had completed their residency within the past two years, revealed a trend that intermediates between the views of professors and residents. Given their transitioning status from learners to educators, their opinions are considered very important. In fact, these opinions represented a compromise between the perspectives of professors and residents in their roles as educators.

Each of the different learning methodologies of ILT and SPL has advantages and disadvantages [15–17]. ILT offers detailed materials and immediate feedback but is limited by structured schedules and instructor variability. SPL allows flexibility in timing and location with the use of open-source materials, enabling repeated review of content, though it lacks the immediate feedback of ILT and may require more time for comprehension. Ultimately, trainees benefit most from actively engaging with the educational material and their learning process.

Although this study focused on resident education, only 14 residents were actually included which is a limitation of this study. However, unfortunately, this is the real-world situation in South Korea. Many doctors are avoiding essential medical services, such as internal medicine, GS, obstetrics and gynecology, and pediatrics. Instead, a majority prefer departments like plastic surgery, ophthalmology, and dermatology, which focus on cosmetic procedures or enhancing the quality of life. This is the reason why, despite being a fairly large-scale national university hospital, the number of residents was small. This situation underlines the importance of further improving the quality of education.

The responses in this study can help standardize the appropriate educational programs for surgical residents in GS departments. Based on these research findings, the authors' institution has decided to enhance technical education with theoretical support. It is currently developing and implementing education on ultrasound and biopsy techniques, laparoscopic bowel anastomosis, and robotic surgery. We established close cooperation with other departments so that in addition to surgical skills training, we could directly experience and learn other departments' techniques (intubation, ventilator manipulation, CPR, and so on.). Various colleagues and companies are supporting this training, viewing it as an investment in the future generation of surgeons. As demonstrated in the authors' study, it is necessary to verify and evaluate the educational programs of each institution for their rationality and efficiency. This approach will contribute to the training of better surgeons by enabling residents to engage more actively in the curriculum.

## Conclusion

The educational impact of training materials and methods can be maximized when surgical residents engage in the preferred training resources that provide them with satisfaction. Therefore, the best educational training course for surgical residents would include providing them with many opportunities to operate and perform surgical procedures themselves. Although written materials and theories remain important, the effect of education is enhanced when the surgical residents’ satisfaction is increased through the provision of practical learning opportunities.

### Supplementary Information

Supplementary Material 1.

Supplementary Material 2.

## Data Availability

The data that support the findings of this study are available from the corresponding author, JHJ, upon reasonable request.
