# Supplementary figures and images for: Importance of multimodal resident education curriculum for general surgeons: perspectives of trainers and trainees
Source: BMC Med Educ. 2024 May 10;24:518. doi: 10.1186/s12909-024-05515-x (PMC11088119; doi:10.1186/s12909-024-05515-x)

## Slide 1
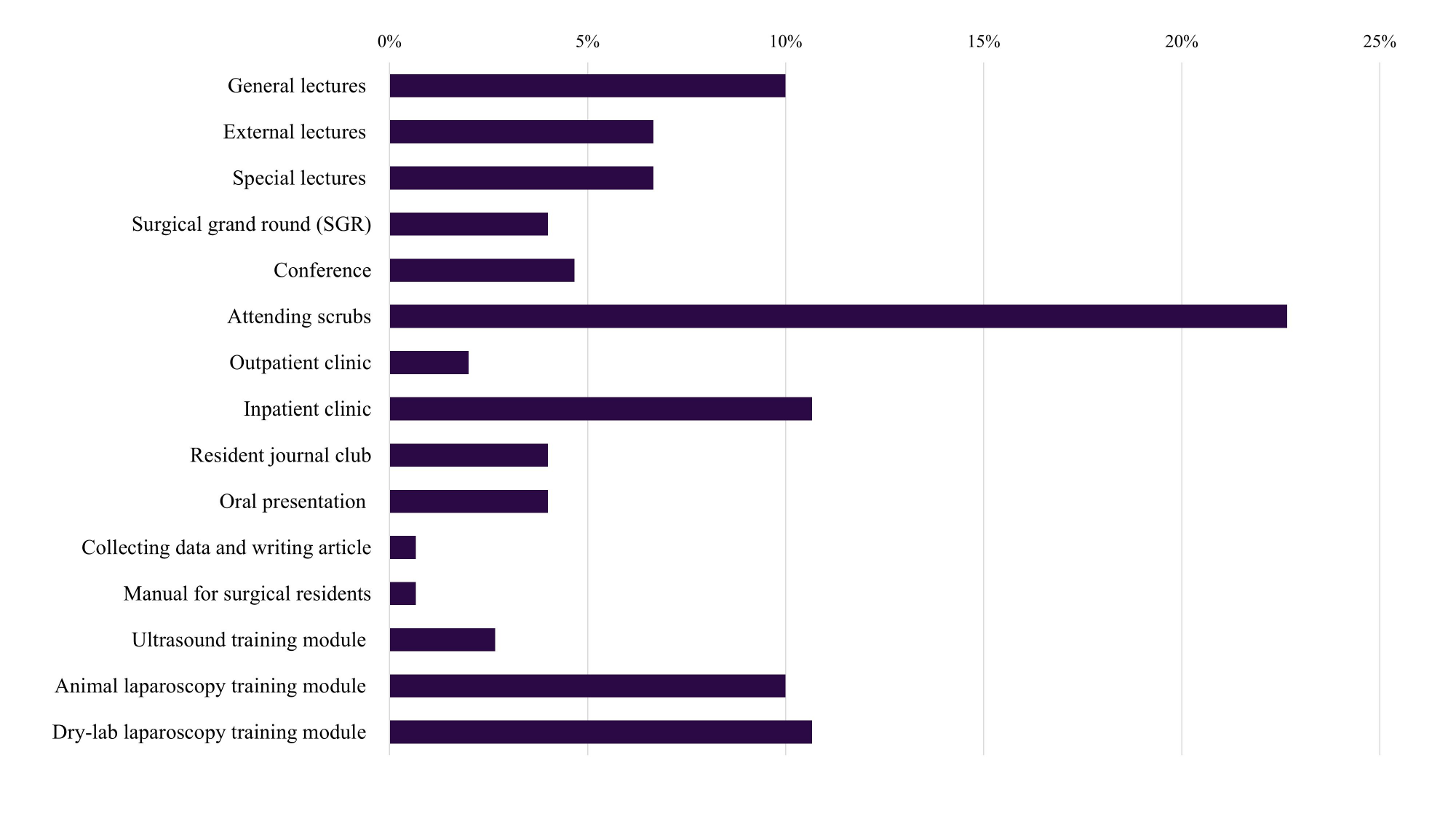

#

Supplement: Supplementary file 1 — Supplementary Material 1. [file 12909_2024_5515_MOESM1_ESM.pptx]
