# Supplementary material for: Importance of multimodal resident education curriculum for general surgeons: perspectives of trainers and trainees
Source: BMC Med Educ. 2024 May 10;24:518. doi: 10.1186/s12909-024-05515-x (PMC11088119; doi:10.1186/s12909-024-05515-x)
Supplement: Supplementary file 2 — Supplementary Material 2. [file 12909_2024_5515_MOESM2_ESM.docx]

Supplementary Table 1. Items of questionnaires

| No. of question | Question | List of answers |
| --- | --- | --- |
| Q1 | What’s your position in the hospital? | 1) First-year resident  2) Second-year resident  3) Third-year resident  4) Clinical fellow  5) Clinical professor  6) Academic professor |
| Q2 | What are your average working hours per day? | 1) 8 hours  2) 10 hours  3) more than 12 hours |
| Q3 | How much time per day do you devote to resident education? | 1) 1 hour  2) 2 hours  3) 3 hours  4) more than 4 hours |
| Q4 | Among the educational curricula, select the three that you think have the most educational effect. | 1) General lectures  2) External lectures  3) Special lectures  4) Surgical grand round (SGR)  5) Conference  6) Attending scrubs  7) Outpatient clinic  8) Inpatient clinic  9) Resident journal club  10) Oral presentation  11) Collecting data and writing an article  12) Manual for surgical residents  13) Ultrasound training module  14) Animal laparoscopy training module  15) Dry-lab laparoscopy training module |
| Q5 | Prioritize the educational category that you think has been most helpful to resident education. | 1) Instructor-led training (ILT)  2) Clinical education  3) Self-paced learning (SPL)  4) Hands-on training |
| Q6 | Prioritize the educational curriculum in the instructor-led training category that you think has been most helpful to resident education. | 1) General lectures  2) External lectures  3) Special lectures  3) Surgical grand round (SGR)  4) Conference |
| Q7 | Prioritize the educational curriculum in the clinical education category that you think has been most helpful to resident education. | 1) Attending scrubs  2) Outpatient clinic  3) Inpatient clinic |
| Q8 | Prioritize the educational curriculum in the self-paced learning category that you think has been most helpful to resident education | 1) Resident journal club  2) Oral presentation  3) Collecting data and writing an article  4) Manual for surgical residents |
| Q9 | Prioritize the educational curriculum in the hands-on training category that you think has been most helpful to resident education. | 1) Ultrasound training module  2) Animal laparoscopy training module  3) Dry-lab laparoscopy training module |

Supplementary table 2. Self-estimated working time and time devoted to the education for resident in a week

|  |  | Professors (n=28) | Fellows (n=8) | Residents (n=14) | |
| --- | --- | --- | --- | --- | --- |
| Mean age (±SD, years) |  | 47.3 ± 15.1 | 33.3 ± 2.3 | 31.0 ± 3.3 | |
| Departments (n, %) | Breast/thyroid | 7 (25.0) | 1 (12.5) | 1^st^ year | 2 (14.3) |
|  | Colorectal | 6 (21.4) | 2 (25.0) | 2^nd^ year | 4 (28.6) |
|  | Upper gastro-intestinal | 5 (17.9) | 1 (12.5) | 3^rd^ year | 8 (57.1) |
|  | Vascular | 4 (14.3) | 3 (37.5) |  | |
|  | Hepato-bilio-pancreatic | 3 (10.7) | - |  | |
|  | Pediatric | 1 (3.6) | - |  | |
|  | Trauma | 1 (3.6) | - |  | |
|  | Critical care | 1 (3.6) | 1 (12.5) |  | |
| Self-estimated working hours per day (n, %) | Mean±SD | 9.9 ± 1.4 | 11.5 ± 0.0 | 11.6 ± 0.0 | |
|  | Less than 8 hours | 8 (28.6) | - | 1 (7.1) | |
|  | 8-10 hours | 13 (46.4) | 2 (25.0) | 1 (7.1) | |
|  | More than 12 hours | 7 (25.0) | 6 (75.0) | 12 (85.7) | |
| Self-estimated time devoted to the education for resident per week (n, %) | Mean±SD | 2.1 ± 0.7 | 2.1 ± 0.7 | 2.1 ± 3.5 | |
|  | Less than 1 hour | 4 (14.3) | 2 (25.0) | 3 (21.4) | |
|  | 1 hour | 6 (21.4) | 3 (37.5) | 4 (28.6) | |
|  | 2 hours | 8 (28.6) | 2 (25.0) | 3 (21.4) | |
|  | 3 hours | 3 (10.7) | - | - | |
|  | More than 4 hours | 7 (25.0) | 1 (12.5) | 4 (28.6) | |
